# Unveiling the molecular architecture of the mitochondrial respiratory chain of *Acanthamoeba castellanii*

**DOI:** 10.15698/mic2025.03.846

**Published:** 2025-03-31

**Authors:** Christian Q. Scheckhuber, Sutherland K. Maciver, Alvaro de Obeso Fernandez del Valle

**Affiliations:** 1 Tecnologico de Monterrey, Escuela de Ingeniería y Ciencias, Ave. Eugenio Garza Sada 2501, 64849, Monterrey, N.L, Mexico.; 2 Centre for Discovery Brain Sciences, Edinburgh Medical School, Biomedical Sciences, University of Edinburgh, Hugh Robson Building, George Square, Edinburgh, EH8 9XD, Scotland, UK.

**Keywords:** Acanthamoeba castellanii, mitochondria, respiratory chain, alternative oxidase, alternative NADH dehydrogenase, uncoupling proteins

## Abstract

* Acanthamoeba castellanii* is a ubiquitous free-living amoeba that can cause severe infections in humans. Unlike most other organisms, *A. castellanii* possesses a "complete" mitochondrial respiratory chain, meaning it con-tains several additional enzymes that contribute to its metabolic versa-tility and survival in diverse environments. This review provides a com-prehensive overview of the mitochondrial respiratory chain in *A. castellanii*, focusing on the key alternative components in-volved in oxidative phosphorylation and their roles in energy metabo-lism, stress response, and adaptation to various conditions. The func-tional characterization of the alternative oxidase (AOX), uncoupling pro-tein (UCP), and alternative NAD(P)H dehydrogenases, highlight their roles in reducing oxidative stress, modulating proton gradients, and adapting to changes in temperature and nutrient availability. These pro-teins and systems serve a role in the survival of *A. castel-lanii* under stressful conditions such as starvation and cold con-ditions. Further knowledge of the respiratory chain of the amoeba has potential implications for understanding the evolution of mitochondrial respiration and developing new therapies for treating *Acanthamoeba* infections.

## Abbreviations

AK - Acanthamoeba keratitis, 

AOX - alternative oxidase, 

BHAM - benzohydroxamate, 

COX - cytochrome-c-oxidase, 

FA - fatty acid, 

FFA - free FA, 

HNE - 4-hydroxy-2-nonenal, 

NAD(P)Halt - alternative NAD(P)H dehyddrogenases, 

ROS - reactive oxygen species, 

UCP - uncoupling protein. 

## INTRODUCTION

*Acanthamoeba castellanii* is a protozoan organism found in various environments worldwide, including soil, water, and air. As an opportunistic pathogen, it can cause a range of infections in humans, including *Acanthamoeba* keratitis (AK), a severe eye infection primarily associated with contact lens wear, and granulomatous amoebic encephalitis (GAE), a rare but often fatal infection of the central nervous system 1,2. The biology of *A. castellanii* involves its ability to exist in two distinct forms: a dormant cyst stage, which is resistant to adverse conditions, and an active trophozoite stage, which feeds on bacteria and other organic matter. This versatility allows *A. castellanii* to survive and thrive in a wide range of habitats 2,3,4,5,6.

**Figure 1:**
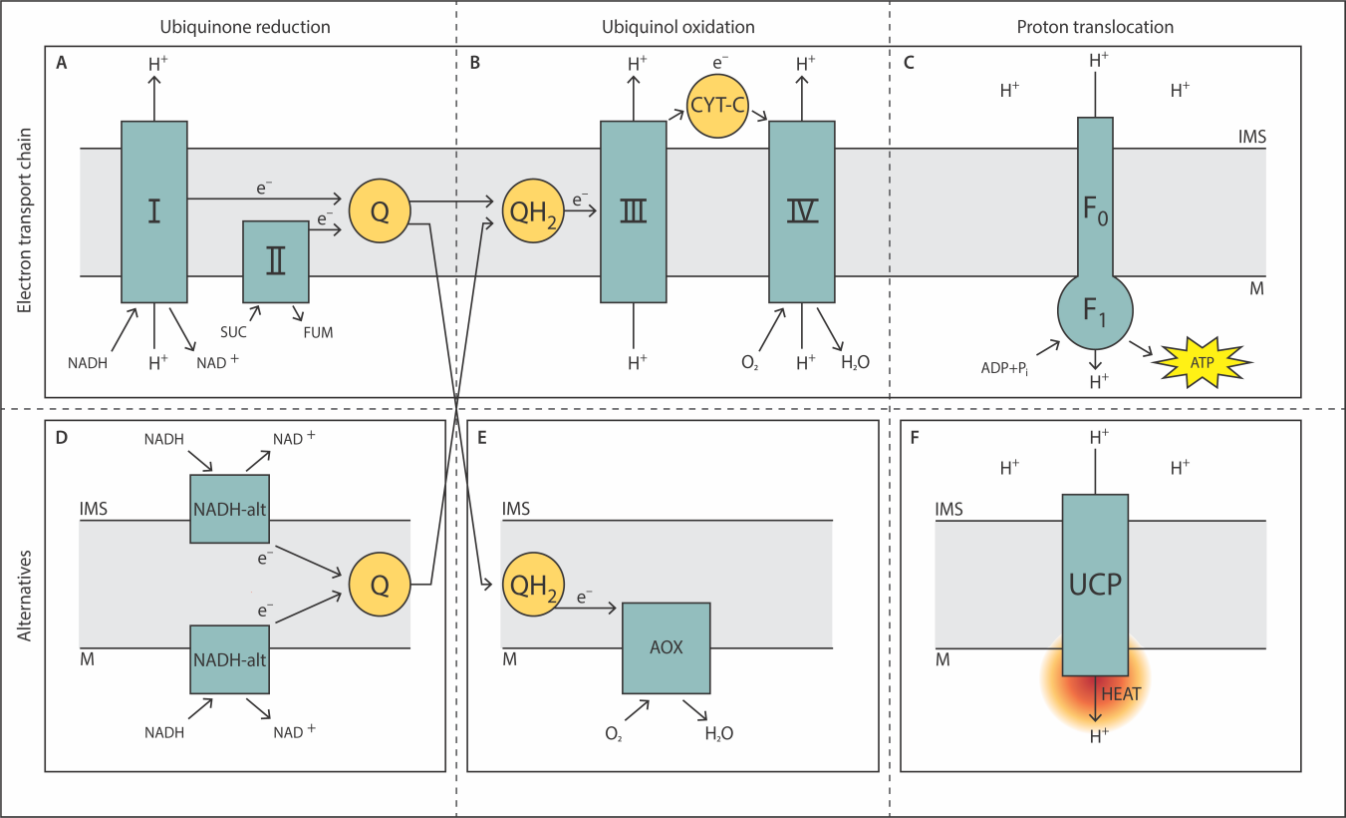
FIGURE 1: Molecular organization of the mitochondrial respiratory chain. The flow of electrons (e^-^) along the mitochondrial membrane and the translocation of protons (H^+^) across the inner mitochondrial membrane is indicated by arrows. The respiratory chain is horizontally divided into segments of ubiquinone reduction, ubiquinol oxidation and proton translocation. **(A)** to **(C)** depict common components of the respiratory chain that can be found in mitochondria of most organisms. **(D)** to **(F)** show alternative components of the respiratory chain that are all found in A. castellanii. AOX: alternative oxidase; CYT-C: cytochrome-c; F_0_: F_0_ subunit of ATP synthase; F_1_: F_1_ subunit of ATP synthase; FUM: fumarate; I: NADH:ubiquinone oxidoreductase; II: succinate-coenzyme Q reductase; III: coenzyme Q: cytochrome-c-oxidoreductase; IV: cytochrome-c oxidase; IMS: intermembrane space; M: matrix; NADH-alt: alternative NADH dehydrogenase; Q: ubiquinone; QH_2_: ubiquinol; SUC: succinate; UCP: uncoupling protein.

Intriguingly, in contrast to most other organisms, *A. castellanii* is characterized by possessing a “complete” mitochondrial respiratory chain, meaning that it contains several additional enzymes such as alternative NADH dehydrogenases 7, alternative oxidase (AOX) 8,9,10, and uncoupling protein (UCP) 11 along with the common components of the electron transport chain (complex I to IV and F_0_F_1_ ATP synthase) that are a hallmark of mitochondria from most organisms (**Figure 1A-C**). This adaptability could contribute to allowing *A. castellanii* to escape medical treatments aimed at treating infections and surviving adverse environmental conditions. In the following sections, these additional proteins, along with their functions, are described.

The mitochondrion, as the organelle of oxidative phosphorylation, is the main source of reactive oxygen species (ROS) in aerobic heterotrophic organisms 12. ROS intermediates such as superoxide, hydrogen peroxide and hydroxyl radicals among several other types may arise during cytochrome-*c*-oxidase (COX)-dependent respiration as by-products 13. The respiration chain found mainly in the mitochondria to produce ATP has been reviewed, for more information which goes beyond the scope of this article the reader is referred to reviews previously published 14,15,16. Cellular respiration occurs through several different mechanisms depending on the organism, the environment, the conditions of the cell or other conditions. The most common configuration includes five different enzymatic complexes (I through IV and ATP synthase) and two different electron carriers (**Figure 1A-C**). Complexes I, III and IV generate a proton gradient and transport electrons that mainly results in the production of ATP. The electron carriers are hydrophobic ubiquinone (Q) and hydrophilic cytochrome-*c* which transfer electrons between the different enzymatic complexes 16. Putting this into perspective, the electron carrier ubiquinone is reduced to ubiquinol (QH_2_) by NADH or succinate dehydrogenase activity. Usually, two electrons of QH_2_ are transferred individually to cytochrome-*c* reductase (complex III of the respiratory chain), which has two electron acceptors (cytochrome-bc1 and the "Rieske" iron-sulfur protein). These single electron transfer steps pose a risk for increasing the production of ROS because superoxide anions might be formed. COX is the common terminal oxidase of the respiratory chain leading to the reduction of oxygen to form water. Mitochondria have different adaptative tools that help maintain cell homeostasis and organismal survival in changing conditions. These mechanisms are regulated by different processes which include gene expression, transmembrane transport, enzymatic activities, protein complex formation and metabolite levels 17. Many of these adaptations can be found in *Acanthamoeba* such as AOX which are capable of bypassing complexes III and IV, UCPs and alternative NAD(P)H dehydrogenases (NAD(P)H*alt*) (**Figure 1D-F**). All these proteins which can be found in *Acanthamoeba* are the focus of this review and a quick summary of each component can be found in **Table 1** along with a diagram explaining the process in **Figure 1**.

**Table 1:** Summary of components of the mitochondrial respiratory chain.

**Name**	**Organization**	**Main Functions**	**Other names**
Complex I	Enzymatic complex	Oxidizing NADH, pumping four protons and producing ubiquinol	Type I NADH dehydrogenase, NADH:ubiquinone oxidoreductase
Complex II	Enzymatic complex	Oxidizing succinate to fumarate and producing ubiquinol	Succinate dehydrogenase, succinate-coenzyme Q reductase
Complex III	Enzymatic complex	Oxidizing ubiquinol, reducing cytochrome-*c* and pumping four protons	Coenzyme Q: cytochrome-*c*-oxidoreductase, cytochrome* bc1* complex
Complex IV	Enzymatic complex	Oxidizing cytochrome-*c* and reducing oxygen to water and pumping 2 protons	Cytochrome-*c *oxidase
ATP synthase	Enzymatic complex	Synthetizing ATP by using the proton gradient	Complex V, F_0_F_1_ ATP synthase
Alternative oxidase	Protein	Oxidizing ubiquinol and reducing oxygen to water	AOX
Uncoupling protein	Protein	Hydrophobic proton channel through the inner mitochondria membrane	UCP
Alternative NADH dehydrogenase	Protein	Oxidizing NADH and producing ubiquinol	NAD(P)H*alt*, alternative NADH:ubiquinone oxidoreductase
Ubiquinone	Low molecular weight compound	Hydrophobic electron carrier from Complex I (or NADH*alt*) and II to Complex III (or AOX)	Q, and when reduced ubiquinol, QH_2_
Cytochrome-*c*	Protein	Hydrophilic electron carrier from Complex III to IV	Cyt *c*

## ALTERNATIVE OXIDASE - OXIDIZING QH_2_ IN PLACE OF COMPLEX III

In some organisms, an AOX exists that can directly transfer electrons from QH_2_ to molecular oxygen, thus bypassing complex III and COX (**Figure 1E**). AOX is not a heme-containing protein like COX but instead belongs to the group of di-iron carboxylate proteins 18. In these proteins, the anionic side chains of the amino acids change their spatial position by reducing the iron center and thus provide several access points for oxygen to the active site of the AOX 19. This multiple coordination is important for oxygen splitting. Inhibitors of COX such as cyanide and azide cannot effectively inhibit AOX 20. However, hydroxy-amino acids (R-CONHOH), such as salicylic hydroxamic acid (SHAM), do inhibit AOX 21. AOX is a potential target for combatting *A. castellanii*, therefore we explore its characteristics in this section in detail. In the following chapter we first give an overview of the structure and function of AOX in *A. castellanii*. We then summarize studies that highlight the tasks of AOX within the context of the respiratory chain and continue with the role of AOX-dependent respiration to control oxidative stress levels inside the cell. The following subsection discusses means to regulate AOX, such as allosteric and transcriptional regulation. In the last subsection of this chapter, we summarize the contents and give an outlook on AOX in other amoebae.

### AOX - functions and structural analyses

Two hypotheses have been put forward for the function of AOX. The first hypothesis states that AOX enables the continuation of essential dissimilatory processes such as glycolysis and the citric acid cycle in the case of COX inhibition (or lack of ADP which limits the activity of the F_0_F_1_-ATP-synthase) 22,23. The second hypothesis is that less ROS is produced during AOX-dependent respiration 24,25. AOX gene expression has been shown to be induced by cellular stress in plants 20,26. As stated previously, when QH_2_ formed by NADH dehydrogenase is oxidized by AOX, two of the three proton pumps of the respiratory chain are bypassed (complex III and COX). This results in a lowered membrane potential and decreased ATP synthesis by ATP synthase 27.

In addition to AOX from numerous plants 26,28, corresponding proteins have been demonstrated to exist in fungi 29, and isolated from protists such as the causative agent of sleeping sickness *Trypanosoma brucei* 30 and *A. castellanii* 8. In the latter, two different AOX genes were subsequently identified and labeled AOX isoform A and B 10.

Although AOX had been described earlier in *A. castellanii* 31, the first immunological demonstration of the enzyme was achieved in 1997. Monoclonal antibodies against AOX from voodoo lily (*Sauromatum guttatum*, family Araceae) could detect several forms of AOX in *A. castellanii* which has three monomeric forms (38, 35, and 32 kDa) and very low amounts of a single 65 kDa dimeric form 8. This result holds special significance in an evolutionary context, as there is speculation that protozoan amoebae could serve as a pivotal divergence point for higher fungi, plants and animals 32, with the latter lacking the AOX. Interestingly, the analyses pointed out that while in plants AOX occurs mostly as a dimer, in *A. castellanii* it functions as a monomer. The authors could also observe time-dependent changes with the age of amoeba cell culture: the level of the monomeric 35 kDa form declines with the aging of the culture (78 h) following a peak at 24 h. In a similar fashion, AOX-dependent respiration reaches its peak at 24 h and declines thereafter over the course of three days. This observation is crucial as it shows that AOX biosynthesis, assembly and functionality are changing during the developmental phases of the amoebae which might exhibit different energetic demands.

### Functional characterization of the AOX-dependent respiration in *A. castellanii*

As mentioned before, *A. castellanii*’s mitochondria have two pathways for quinol oxidation: the COX pathway and the AOX pathway. There are several ways to quantify mitochondrial activity, one of which is measuring the mitochondrial ADP/O ratio which refers to the ratio of the amount of adenosine diphosphate (ADP) phosphorylated to ATP to the amount of oxygen consumed during oxidative phosphorylation in mitochondria. This ratio reflects the efficiency of oxidative phosphorylation in coupling the consumption of oxygen to the synthesis of ATP. In other words, it indicates how many molecules of ATP are synthesized per molecule of oxygen consumed during mitochondrial respiration. A higher ADP/O ratio indicates greater efficiency in ATP production per unit of oxygen consumed. Using this approach, researchers assessed each pathway's contribution to phosphorylating state 3 respiration in isolated mitochondria treated with guanosine mono phosphate (GMP, activator of AOX) and succinate as an oxidizable substrate for the respiratory chain 33. This study was the first to explore the kinetics of both pathways concurrently in *A. castellanii*, shedding light on electron distribution under varying quinone-reducing rates. Results showed that as overall respiration decreased after adding the complex II inhibitor malonate, the alternative pathway contribution declined sharply below 50% quinone reduction, thus becoming negligible, while the cytochrome pathway peaked at 58% before eventually declining. The authors concluded that AOX-dependent respiration accounts for approximately 40% under the GMP-stimulated conditions.

The activity of *A. castellanii* mitochondria's two quinol-oxidizing pathways, the phosphorylating cytochrome pathway, and the AOX, has been determined by measuring the ubiquinone (Q) pool redox state, regardless of the reducing substrate 34. Both pathways exhibit increased activity (up to 80%) when Q reduction (Q_reduced_/Q_total_) levels increase. However, cytochrome pathway activity decreases by about 50% when Q reduction levels exceed 80%, likely due to limitations of the Q cycle mechanism of complex III. These data suggest that AOX might help as a kind of electron overflow protection system, thus minimizing the probability of superoxide anion production at complex III. This would potentially endow *A. castellanii* with an elevated resistance against compounds that act via elevating intracellular oxidative stress, such as methyl viologen (also known by its commercial name Paraquat).

As outlined above, AOX can help with the maintenance of temperature inside the cell. There are interesting data that show that AOX biosynthesis positively correlates with decreases ambient temperature. For example, when grown temporarily at low temperatures, *A. castellanii* mitochondria showed higher oxygen consumption but lower coupling parameters, i. e., respiratory control ratio (phosphorylating oxygen consumption, state 3, to non-phosphorylating oxygen consumption, state 4) and ADP/O values, compared to those grown at normal temperatures 35, which means a lowered efficiency of ATP biosynthesis. However, the presence of the AOX inhibitor benzohydroxamate (BHAM) equalized respiratory rates and coupling parameters, suggesting the cytochrome pathway remained intact despite cold growth conditions. ADP/O measurements confirmed an increased contribution of the alternative oxidase to total mitochondrial respiration in cold-grown cells. Additionally, mitochondria from cold-grown cells exhibited higher levels of the AOX, correlating with increased cyanide-resistant respiratory activity, both stimulated and unstimulated by GMP. AOX has also been related to thermogenesis and studied in other protsits such as *T. brucei* 36. Collectively, these findings indicate that AOX helps *A. castellanii* to survive low temperatures in its environment.

### AOX-dependent respiration and the redox status of the Q pool in relation to oxidative stress

One of the most intriguing aspects of AOX-dependent respiration is its potential to mitigate oxidative stress. In this line of investigation, several studies have been conducted which are briefly summarized here. Importantly, the energetic characteristics of *A. castellanii* mitochondria respiring with malate as substrate, as well as their response to oxidative stress induced by hydrogen peroxide in the presence of Fe^2+^ ions were extensively characterized 37. Surprisingly, contrary to previous observations 38, the investigation revealed that mitochondria treated with H_2_O_2_ did not exhibit significant impairment. Notably, no substantial alterations in cytochrome pathway activity, as evidenced by the absence of effects on uncoupled and phosphorylating respiration rates and coupling parameters upon inhibition of the alternative oxidase were observed. However, in the absence of an AOX inhibitor, non-phosphorylating respiration declined progressively with increasing H_2_O_2_ concentration, while coupling parameters such as the respiratory control ratio and ADP/O ratio showed slight improvement, suggesting potential inactivation of AOX. Additionally, no discernible changes in membrane potential, Ca^2+^ uptake and accumulation capacity, mitochondrial outer membrane integrity, or cytochrome-*c* release between mitochondria treated with 0.5-25 mM H_2_O_2_ and control (untreated) mitochondria could be measured. These data collectively indicate that brief (i.e., a few minutes) exposures of *A. castellanii* mitochondria to H_2_O_2_ in the presence of Fe^2+^ do not compromise their fundamental energetic functions. This result does not rule out that more long-term exposures of *A. castellanii* to H_2_O_2_ have measurable effects on the mitochondrial function. Consequently, treating *A. castellanii* cell cultures at various growth stages with a moderate concentration (1.4 mM) of H_2_O_2_ elicited distinct responses to oxidative stress 39. Remarkably, treatment with H_2_O_2_ during exponential growth phases significantly impeded cell proliferation; however, mitochondrial function remained unscathed in isolated mitochondria from these cells. Conversely, exposure to H_2_O_2_ as cells approached the stationary phase exerted negligible impact on growth and viability but severely compromised mitochondrial bioenergetic function. Despite the preservation of mitochondrial integrity, oxidative damage manifested in diminished cytochrome pathway activity, uncoupling protein function, oxidative phosphorylation efficiency, as well as reductions in membrane potential and endogenous ubiquinone levels during resting states. Intriguingly, late H_2_O_2_ exposure prompted an upsurge in AOX protein levels and activity, alongside augmented membranous ubiquinone content in isolated mitochondria. This marked the first demonstration of the regulatory role of ubiquinone content within the inner mitochondrial membrane in response to oxidative stress. This might also constitute a strategy for surviving conditions of elevated ROS production, such as during treatment of keratitis by drugs.

The relationship between the rate of mitochondrial ROS formation and the reduction level of mitochondrial Q pool across varying degrees of engagement of key mitochondrial pathways using mitochondria isolated from the amoeba *A. castellanii* shed light on the relationship of mitochondrial redox status and oxidative stress 40. Significant shifts in the Q pool's reduction state, achieved by selectively inhibiting QH_2_-oxidizing pathways and modulating the oxidative phosphorylation system were achieved. A direct correlation between ROS formation and the reduction level of the Q pool across both QH_2_-oxidizing pathways was observed. Intriguingly, a higher Q reduction level was found to correspond to increased ROS generation. For the cytochrome pathway, ROS production exhibited a nonlinear dependency on the Q reduction level, with a pronounced impact observed at values surpassing the Q reduction level of the phosphorylating state (~ 35%). Furthermore, as the Q pool's reduction level escalated beyond 40%, AOX-dependent respiration is induced, particularly in response to heightened ROS production via the cytochrome pathway. The authors proposed that the redox status of the Q pool serves as an endogenous indicator (endogenous Q redox state), and consequently a valuable tool for indirectly assessing overall ROS production in mitochondria. This result is also of practical importance, as it allows to specifically measure the efficiency of ROS-altering compounds to combat the amoebae.

### AOX regulation by purine nucleotides and transcriptional aspects

Affecting the activity of an enzyme can not only be achieved by targeting its catalytic function but also by finding ways to interfere with its regulation. As mentioned above, the activity of *A. castellanii* AOX is stimulated by GMP 33. An early study highlights the pH dependence of the AOX activity, with an optimum pH of 6.8, regardless of the reducing substrate 41. GMP stimulation of AOX is strongly influenced by pH, likely involving protonation/deprotonation processes. The redox state of ubiquinone also affects AOX activity, with the highest activity observed at pH 6.8. These observations suggest that pH, GMP binding, and ubiquinone redox state collaboratively regulate the activity of AOX in *A. castellanii* mitochondria. Furthermore, the high pH sensitivity of AOX may link inactivation of cytochrome pathway proton pumps to AOX activation, potentially leading to accelerated redox free energy dissipation, thereby generating heat.

Previous research revealed that guanine nucleotides exhibit a more pronounced activation of AOX compared to adenine nucleotides 42. Interestingly, nucleoside monophosphates emerged as more efficient activators than their di- or triphosphate counterparts. The magnitude of nucleotide influence on AOX activity was contingent upon the medium's pH, with maximal impact observed at pH 6.8, coinciding with the optimal activity of AOX. Importantly, ATP was found to exert an inhibitory effect on AOX activity, sharply contrasting with other purine nucleotides. This inhibition by ATP was not exclusive to *A. castellanii* mitochondria but was also observed in other protozoa such as *Dictyostelium discoideum* and yeast like *Candida maltosa*, suggesting a potentially common regulatory feature among purine nucleotide-modulated non-plant AOXs. Kinetic analysis unveiled that the binding of GMP, a positive allosteric effector, and ATP, a negative allosteric effector, to AOX are mutually exclusive. ATP's inhibition can be counteracted by sufficiently high concentrations of GMP, and vice versa. Notably, GMP exhibits a half-maximal effect on AOX activity at approximately three times lower concentrations compared to ATP, indicating a higher binding affinity for the positive effector. It is suggested that AOX activity in *A. castellanii* mitochondria may be intricately modulated by the relative intracellular concentrations of purine nucleotides.

Transcriptional regulation of AOX gene expression has been studied throughout the growth phases of amoeba batch culture by using qPCR 42. It was found that AOX transcript levels were highest in trophozoites that were cultivated for 12 h. After this point there was a steady decline in transcript and protein levels of AOX. Due to the minuscule difference between AoxA and AoxB (12 bp insertion in the targeting sequence) the two forms were not differentiated so they both were measured together. Additionally, AOX was introduced into AOX-deficient *Escherichia coli* to study its behavior in the prokaryote. *E. coli* harbors quinol oxidases of the bo and bd type in its plasma membrane and after AOX introduction exhibited cyanide-resistant BHAM-sensitive respiration which is a testament to the versatility and efficacy of AOX even within a foreign host environment. AOX activity in the transformed bacteria responded dynamically to purine nucleotides—a phenomenon reminiscent of its behavior in the mitochondria of *A. castellanii* outlined above. The stimulation by GMP and inhibition by ATP again underscored a sophisticated regulatory interplay, wherein AOX activity is intricately modulated by the mutual exclusion of purine nucleotides. A later study used RNAseq to determine transcript levels of respiratory chain related genes and showed that both AOX isoforms are down-regulated 24 h after encystation initiation but up-regulated after 72 h 43, which might be vital for maintaining a physiological ROS balance.

### AOX in other amoebae and summary

Not only in *A. castellanii* has the alternative respiratory pathway been the subject of dedicated research. Large free-living amoebae, such as *Chaos carolinensis*, exhibit remarkable survival capabilities in spring water even without food intake for extended periods, spanning several weeks 44. This endurance through starvation triggers a notable transformation in mitochondrial cristae morphology, transitioning from a random tubular structure to an ordered (paracrystalline) cubic form. To monitor the metabolic changes accompanying fasting, whole-cell polarography was employed, revealing a progressive decrease in basal respiration per cell alongside an increase in the AOX. Additionally, spectrofluorometric analysis of cell lysates using 2',7'-dichlorofluorescein diacetate highlighted a heightened generation of H_2_O_2_ and ROS in starved cells compared to fed counterparts 44. Intriguingly, fluorescence microscopy of intact cells incubated with the same dye showcased the accumulation of H_2_O_2_ and ROS within vacuoles. A remarkable observation was the significant generation of O_2_ observed in starved cells following the addition of KCN. This phenomenon may be attributed to the release of H_2_O_2_ from vacuoles into the cytosol, where it can interact with catalase 44. Collectively, these indications underscore the increased oxidative stress experienced by amoebae during fasting and illuminate the organism's arsenal of protective mechanisms to mitigate it. Notably, activation of a plant-like alternative oxidase is suggested as one such protective measure. Furthermore, the hypothesis is proposed that the cubic structural transition of the mitochondrial inner membrane serves as an additional safeguard, mitigating oxidative damage by facilitating the efflux of H_2_O_2_ and ROS while concurrently reducing the susceptibility of membrane lipids to oxidative agents.

## UNCOUPLING PROTEIN - REGULATOR OF CATABOLISM AND OXIDATIVE STRESS

In addition to AOX, UCPs contribute to the adaptability of the mitochondrial respiratory chain to changing conditions. UCPs are mitochondrial transport proteins involved in the regulation of organismal energy metabolism 45,46. Like the components of the respiratory chain, UCPs are located in the inner mitochondrial membrane and play a crucial role in generating heat by dissipating the proton gradient generated by oxidative phosphorylation (**Figure 1F**). The phenomenon known as proton leak lowers the efficiency of ATP production and releases energy as heat. This is of high importance in processes such as thermogenesis in brown adipose tissue in mammals. There are several types of UCPs, including UCP1, UCP2, and UCP3, each with distinct tissue distributions and functions 47. In mammals, UCP1 is primarily responsible for non-shivering thermogenesis in brown fat, while UCP2 and UCP3 are more broadly expressed and have roles in regulating metabolic efficiency and reducing reactive oxygen species. A UCP has also been detected and characterized in *A. castellanii*. It was found to be the first cold-response protein in a unicellular organism 48. Growing *A. castellanii* in batch culture at a temperature of 6°C instead of 28°C led to a substantial increase in UCP content along with an increase in state 4 respiration. This might help the amoeba to survive adverse conditions of low temperature in the wild. Mechanistically, it was shown in *A. castellanii* that UCP shifts energy from phosphorylating state 3 respiration in a manner that is dependent on fatty acids (FA) such as linoleic acid 49, linking mitochondrial respiration and FA metabolism. In a subsequent study, different FA were tested for their effects on UCP activity 50. The authors showed that the most effective uncouplers of oxidative phosphorylation were unsaturated FA such as linoleic (C18:2) and oleic acid (C18:1). These could reduce the respiratory control ratio from 2.42 (control, no FA) to 1.05 and 1.18, respectively. This means that the efficiency of ATP biosynthesis is significantly decreased. It should be kept in mind that the availability and composition of FA changes during the growth and development of the cells. FA-mediated regulation of mitochondrial respiration might constitute a way for the amoeba to adapt their energy metabolism to changing demands.

The links between FA, mitochondrial respiration and ROS production have been studied in mitochondria extracted from *A. castellanii* by comparing the free fatty acid (FFA)-activated, purine nucleotide-inhibited UCP, and the FFA-insensitive, purine nucleotide-activated ubiquinol AOX, in mitigating ROS production 11. The study revealed that the activation of UCP through externally introduced FFAs led to a substantial reduction in H_2_O_2_ production. Conversely, inhibition of FFA-induced UCP activity by GDP or the addition of bovine serum albumin (BSA) amplified H_2_O_2_ generation. Similarly, stimulation of antimycin-resistant AOX-mediated respiration by GMP markedly diminished H_2_O_2_ production, whereas inhibition of the oxidase by BHAM negated the GMP-induced decrease in H_2_O_2_ levels. When both energy-dissipating systems were concurrently active, they exhibited a synergistic effect, further decreasing H_2_O_2_ formation. These results strongly suggest that safeguarding against mitochondrial oxidative stress may constitute a vital physiological function of AOX and UCP in unicellular organisms, exemplified by *A. castellanii*.

Another way to boost UCP activity in *A. castellanii* is by adding 4-hydroxy-2-nonenal (HNE) to isolated mitochondria 51. HNE is an end-product of lipid peroxidation and an established marker of oxidative stress 52. By inducing a UCP mediated proton leak HNE can decrease the yield of oxidative phosphorylation and consequently ATP production. Interestingly, HNE-induced UCP can be inhibited by GTP only when the hydrophobic electron-carrier Q is sufficiently oxidized. This suggests that the sensitivity of UCP to GTP inhibition depends on the redox state of Q in active, phosphorylating mitochondria. In summary, this study hints to a function of UCPs in limiting mitochondrial ROS production by a negative feedback loop involving HNE as a messenger molecule.

A recent study described the heterologous expression of the *A. castellanii* coding gene for UCP in baker´s yeast, *Saccharomyces cerevisiae*, provided functional evidence that it encodes a mitochondrial protein with uncoupling activity and that it can decrease elevated superoxide levels in a deletion mutant of the mostly cytosolic superoxide dismutase 1 53,54. UCP in *A. castellanii* are more challenging to target than AOX, because humans do possess UCP, unlike AOX. As such, comparative structural studies of UCP from both organisms are essential to help identifying drugs that target only the microbial UCPs but not the human homologs.

## ALTERNATIVE NAD(P)H DEHYDROGENASES - EXPANDING THE FUNCTIONALITY OF THE RESPIRATORY CHAIN

In addition to AOX and UCP, respiratory chains may contain internal and/or external NAD(P)H*alt* which have not been identified in human mitochondria (**Figure 1D**). Internal refers to the location inside the mitochondrial matrix side of the inner mitochondrial membrane while external means the outer side of the membrane, facing the intermembrane space of mitochondria. NAD(P)H*alt* are usually small proteins (approximately 50 to 60 kDa) that contain FAD or FMN as cofactors that aid in electron transfer reactions. The electron acceptor is ubiquinone. In contrast to complex I of the respiratory chain, NAD(P)H*alt* does not pump protons across the inner mitochondrial membrane. They have been suggested to have the following functions: (a) diminishing ROS formation by energy dissipation, (b) decreasing excess reducing equivalents (recycling the oxidized forms so that glycolysis etc. can continue), (c) some data indicate that NAD(P)H*alt* could increase ROS production, causing apoptosis in some fungi and protists 55,56, (d) in general they seem to perform redox balancing or sensing and (e) act as "energy boosters" supporting developmental transitions (as shown in the intracellular parasite *T. brucei*) by rapidly oxidizing NADH that was formed in the TCA cycle. The external NAD(P)H*alt* of *A. casteIlanii* have been biochemically characterized 57: the coupling parameters, ADP/O and the respiratory control ratio for external NADH and NADPH oxidation were similar, indicating comparable efficiencies in ATP synthesis. Both processes had an optimal pH of 6.8, independent of the ubiquinol-oxidizing pathways, cytochrome pathway, or GMP-stimulated AOX. The maximal oxidizing activity for external NADH was nearly double that of external NADPH. However, external NADPH oxidation had a lower Michaelis constant (K_M_) compared to external NADH oxidation. Additionally, stimulation by Ca^2+^ was approximately ten times higher for external NADPH oxidation, whereas NADH dehydrogenase(s) showed only slight dependence on Ca^2+^.

Importantly, genes encoding NAD(P)H*alt* and AOXs are co-expressed, as shown in microarray and RNAseq data for different stress and developmental conditions 43,58,59,60. In these scenarios, complex I might function as the main proton pump. Lastly, NAD(P)H*alt* activity links respiratory chain to glutathione metabolism (via regeneration of the glutathione reductse enzyme) which has recently been shown to be relevant for the encystment of *A. castellanii* and which is also connected to handling oxidative stress inside the cells 61.

## CONCLUSION - INCREASING OUR UNDERSTANDING OF THE RESPIRATOrY CHAIN FOR NEW TREATMENT OPTIONS

The respiratory chain in *Acanthamoeba* is one of the most complete that has been described. Few other organisms are known to have this wide range of alternatives to adapt mitochondrial function to the needs of the cell and produce ATP 43. These mechanisms provide *Acanthamoeba* with options to survive in the environment and combat treatments during infection. One such adaptation is its ability to survive in low oxygen conditions which allows the amoeba to travel through metabolically active biofilms from which they graze 62. The "brain-eating amoeba" *Naegleria fowleri* has genetic tools to survive under anaerobic conditions through the β-oxidation of FAs into Acetyl-CoA which can then be turned into ATP 63. *Acanthamoeba* could have similar tools that are worth exploring. On the other hand, *Acanthamoeba*’s growth can be inhibited by high-oxygen concentration. High oxygen leads to increased ROS production and oxidative stress which affects the growth of the amoeba 64. Alterations in the respiratory chain are linked to disease and therefore may lead to cell death.

It needs to be mentioned that mitochondrial regulatory mechanisms must ensure that certain combinations of respiratory components do not coincide. An example is the hypothetical situation in which alternative NADH dehydrogenases operate, but not complex I. If, under this condition, AOX is strongly active, ubiquinol would still be oxidized, but no proton gradient across the inner mitochondrial membrane would be generated, as the alternative components cannot translocate protons across the IMS (**Figure 1D, E**). This might lead to ATP depletion because the F_0_F_1_-ATP-synthase would reverse its operation to act as an ATPase that pumps protons into the IMS to maintain basic mitochondrial transport functions. Genetic or pharmacological elicitation of respiratory dysregulation could be a promising approach to combat *A. castellanii* and the severe infections it causes.

*Acanthamoeba* can cause two principal types of infection: *Acanthamoeba* keratitis and granulomatous amoebic encephalitis. However, it has also been reported to cause other infections, mainly in immunocompromised patients such as cutaneous acanthamebiasis 65, and *Acanthamoeba* rhinosinusistis 66,67. These infections do not have many specific treatment options and tend to have a poor prognosis. Mitochondria have been suggested as attractive drug targets against certain infections, such as the ones caused by trypanosomatids, since the impairment of certain proteins of mitochondria can trigger apoptosis and thus cause death of the pathogen 68. Also, as mitochondria serve important metabolic functions such as producing diverse cellular compounds and mediating cell signaling through ROS production and detoxification, they are targets of choice for several bacterial organisms that focus their attacks on the organelle, just like therapies could do 69. Unfortunately, *Acanthamoeba* mitochondria, having a wider range of alternatives to compensate for dysfunction, might prove to be harder challenges for treatment as there is a high probability that this could affect the patient's own mitochondria and thus their cells more severely. As *Homo sapiens* do not have these alternatives in their mitochondrial respiratory chain, AOX and NAD(P)H*alt* could be potential targets for treatment of *Acanthamoeba*-mediated pathologies 43. For example, identifying ways to overexpress both AOX and NAD(P)H*alt* encoding genes could decrease energy availability inside the amoeba by decreasing the proton gradient needed for ATP biosynthesis (AOX and NAD(P)H*alt* do not pump protons, **Figure 1D, E**).

It should be mentioned that *Acanthamoeba* can also act as a reservoir for other microorganisms that use it as a "trojan horse" to invade their hosts and to survive in the new environment. Some of these include *Legionella pneumophila* 70,71, *Coxiella burnetti* 72; *Vibrio cholerae* 73, adenoviruses 74 and *Cryptococcus neoformans* 75. In many cases, the relationship between the amoeba and the endosymbiont can increase their pathogenicity and virulence 76,77,78. Finding the metaphorical Achilles heel of the *Acanthamoeba* respiratory chain could not only help control infections of amoebic origin, but also limit some bacterial, viral and fungal outbreaks.

Understanding the respiratory chain in *Acanthamoeba *in its entirety could help us appreciate general mechanisms of metabolic adaptation and therefore develop treatments with a higher chance of success. Additionally, having COX, AOX, NAD(P)H*alt* and UCP could help to understand mitochondrial respiration in many other cell types. *Acanthamoeba* provides a resilient, easily cultured option with a complete respiratory chain that is difficult to find in other organisms. Further studies into the respiratory chain of *Acanthamoeba* could help elucidate the respiratory chain in a wide range of organisms, helping to develop new therapies against AK or even provide insights into the evolution of the mitochondria in general and the respiratory chain in particular.

## CONFLICT OF INTEREST

The authors declare no competing interest.

## References

[1] Marciano-Cabral F, Cabral G (2003). Acanthamoeba spp. as agents of disease in humans.. Clin Microbiol Rev.

[2] Lorenzo-Morales J, Khan NA, Walochnik J (2015). An update on Acan-thamoeba keratitis: diagnosis, pathogenesis and treatment.. Parasite.

[3] Lloyd D (2014). Encystment in Acanthamoeba castellanii: A review.. Exp Parasitol.

[4] Griffiths AJ, Hughes DE (1969). The physiology of encystment of Hart-mannella castellanii*.. J Protozool.

[5] Neff RJ, Ray SA, Benton WF, Wilborn M (1964). Chapter 4 Induction of synchronous encystment (differentiation) in Acanthamoeba sp..

[6] Geisen S, Fiore-Donno AM, Walochnik J, Bonkowski M (2014). Acan-thamoeba everywhere: High diversity of Acanthamoeba in soils.. Parasitol Res.

[7] Joseph-Horne T, Hollomon DW, Wood PM (2001). Fungal respiration: a fusion of standard and alternative components.. Biochimica et Biophysica Acta.

[8] Jarmuszkiewicz W, Wagner AM, Wagner MJ, Hryniewiecka L (1997). Immunological identification of the alternative oxidase of Acanthamoeba castellanii mitochondria.. FEBS Lett.

[9] Vanlerberghe GC (2013). Alternative Oxidase: A Mitochondrial Respiratory Pathway to Maintain Metabolic and Signaling Homeostasis during Abiotic and Biotic Stress in Plants.. Int J Mol Sci.

[10] Henriquez FL, McBride J, Campbell SJ, Ramos T, Ingram PR, Roberts F, Tinney S, Roberts CW (2009). Acanthamoeba alternative oxidase genes: Identification, characterisation and potential as antimicrobial targets.. Int J Parasitol.

[11] Czarna M, Jarmuszkiewicz W (2005). Activation of alternative oxidase and uncoupling protein lowers hydrogen peroxide formation in amoeba Acanthamoeba castellanii mitochondria.. FEBS Lett.

[12] Harman D (1972). The Biologic Clock: The Mitochondria?. J Am Geriatr Soc.

[13] Natvig DO, Sylvester K, Dvorachek WH, Baldwin JL (1996). Superoxide Dismutases and Catalases..

[14] Rich PR, Maréchal A (2010). The mitochondrial respiratory chain.. Essays Biochem.

[15] Tager JM, Wanders RJA, Groen AK, Kunz W, Bohnensack R, Küster U, Letko G, Böhme G, Duszynski J, Wojtczak L (1983). Control of mitochondrial respiration.. FEBS Lett.

[16] Vercellino I, Sazanov LA (2022). The assembly, regulation and function of the mitochondrial respiratory chain.. Nat Rev Mol Cell Biol.

[17] Bennett CF, Latorre-Muro P, Puigserver P (2022). Mechanisms of mitochondrial respiratory adaptation.. Nat Rev Mol Cell Biol.

[18] Andersson ME, Nordlund P (1999). A revised model of the active site of alternative oxidase.. FEBS Lett.

[19] Nordlund P, Eklund H (1995). Di-iron-carboxylate proteins.. Curr Opin Struct Biol.

[20] Elthon TE, Nickels RL, McIntosh L (1989). Monoclonal antibodies to the alternative oxidase of higher plant mitochondria.. Plant Physiol.

[21] Schonbaum GR, Bonner WD, Storey BT, Bahr JT (1971). Specific inhibition of the cyanide-insensitive respiratory pathway in plant mitochondria by hydroxamic acids.. Plant Physiol.

[22] Lambers H (1985). Respiration in Intact Plants and Tissues: Its Regulation and Dependence on Environmental Factors, Metabolism and Invaded Organ-isms..

[23] Vanlerberghe GC, Day DA, Wiskich JT, Vanlerberghe AE, Mclntosh L (1995). Alternative Oxidase Activity in Tobacco Leaf Mitochondria (Depend-ence on Tricarboxylic Acid Cycle-Mediated Redox Regulation and Pyruvate Activation).. Plant Physiol.

[24] Maxwell DP, Wang Y, McIntosh L (1999). The alternative oxidase lowers mitochondrial reactive oxygen production in plant cells.. Proc Natl Acad Sci U S A.

[25] Wagner AM, Moore AL (1997). Structure and function of the plant alternative oxidase: its putative role in the oxygen defence mechanism.. Biosci Rep.

[26] Day DA, Whelan J, Millar AH, Siedow JN, Wiskich JT (1995). Regulation of the alternative oxidase in plants and fungi.. Aust J Plant Physiol.

[27] Czarna M, Sluse FE, Jarmuszkiewicz W (2007). Mitochondrial function plasticity in Acanthamoeba castellanii during growth in batch culture.. J Bio-energ Biomembr.

[28] Siedow JN, Umbach AL (1995). Plant mitochondrial electron transfer and molecular biology.. Plant Cell.

[29] Lambowitz AM, Sabourin JR, Bertrand H, Nickels R, McIntosh L (1989). Immunological identification of the alternative oxidase of Neurospora crassa mitochondria.. Mol Cell Biol.

[30] Clarkson AB, Bienen EJ, Pollakis G, Grady RW (1989). Respiration of Bloodstream Forms of the Parasite Trypanosoma brucei brucei Is Dependent on a Plant-like Alternative Oxidase.. J Biol Chem.

[31] Hryniewiecka L, Jenek J, Michejda JW (1980). Necessity of iron for the alternative respiratory pathway in Acanthamoeba castellanii.. Biochem Bio-phys Res Commun.

[32] Sogin ML, Elwood HJ, Gunderson JH (1986). Evolutionary diversity of eukaryotic small-subunit rRNA genes.. Proc Natl Acad Sci U S A.

[33] Jarmuszkiewicz W, Sluse-Goffart CM, Hryniewiecka L, Michejda J, Sluse FE (1998). Electron Partitioning between the Two Branching Quinol-oxidizing Pathways in Acanthamoeba castellanii Mitochondria during Steady-state State 3 Respiration *.. J Biol Chem.

[34] Jarmuszkiewicz W, Sluse FE, Hryniewiecka L, Sluse-Goffart CM (2002). Interactions Between the Cytochrome Pathway and the Alternative Oxidase in Isolated Acanthamoeba castellanii Mitochondria.. J Bioenerg Biomembr.

[35] Jarmuszkiewicz W, Frączyk O, Hryniewiecka L (2001). Effect of growth at low temperature on the alternative pathway respiration in Acanthamoeba castellanii mitochondria.. Acta Biochim Pol.

[36] Moore AL, Shiba T, Young L, Harada S, Kita K, Ito K (2013). Unraveling the heater: new insights into the structure of the alternative oxidase.. Annu Rev Plant Biol.

[37] Jarmuszkiewicz W, Antos-Krzeminska N, Drachal-Chrul D, Matkovic K, Nobik W, Pieńkowska J, Swida A, Woyda-Ploszczyca A, Budzinska M (2008). Basic energetic parameters of Acanthamoeba castellanii mitochon-dria and their resistance to oxidative stress.. Acta Biochim Pol.

[38] Trocha L, Stobienia O (2007). Response of Acanthamoeba castellanii mitochondria to oxidative stress.. Acta Biochim Pol.

[39] Woyda-Ploszczyca A, Koziel A, Antos-Krzeminska N, Jarmuszkiewicz W (2011). Impact of oxidative stress on Acanthamoeba castellanii mitochondrial bioenergetics depends on cell growth stage.. J Bioenerg Biomembr.

[40] Dominiak K, Koziel A, Jarmuszkiewicz W (2018). The interplay be-tween mitochondrial reactive oxygen species formation and the coenzyme Q reduction level.. Redox Biol.

[41] Jarmuszkiewicz W, Hryniewiecka L, Sluse FE (2002). The Effect of pH on the Alternative Oxidase Activity in Isolated Acanthamoeba castellanii Mitochondria.. J Bioenerg Biomembr.

[42] Antos-Krzeminska N, Jarmuszkiewicz W (2014). Functional expression of the Acanthamoeba castellanii alternative oxidase in Escherichia coli; regu-lation of the activity and evidence for Acaox gene function.. Biochem Cell Biol.

[43] Scheckhuber CQ, Damián Ferrara R, Gómez-Montalvo J, Maciver SK, de Obeso Fernández del Valle A (2024). Oxidase enzyme genes are differen-tially expressed during Acanthamoeba castellanii encystment.. Parasitol Res.

[44] Deng Y, Kohlwein SD, Mannella CA (2002). Fasting induces cyanide-resistant respiration and oxidative stress in the amoeba Chaos carolinensis: implications for the cubic structural transition in mitochondrial membranes.. Protoplasma.

[45] Nicholls DG (2023). Fifty years on: How we uncovered the unique bioener-getics of brown adipose tissue.. Acta Physiologica.

[46] Nicholls DG (2006). The physiological regulation of uncoupling proteins.. Biochim Biophys Acta.

[47] Hirschenson J, Melgar-Bermudez E, Mailloux RJ (2022). The Uncou-pling Proteins: A Systematic Review on the Mechanism Used in the Prevention of Oxidative Stress.. Antioxidants.

[48] Jarmuszkiewicz W, Antos N, Swida A, Czarna M, Sluse FE (2004). The effect of growth at low temperature on the activity and expression of the uncoupling protein in Acanthamoeba castellanii mitochondria.. FEBS Lett.

[49] Jarmuszkiewicz W, Czarna M, Sluse-Goffart C, Sluse F (2004). The contribution of uncoupling protein and ATP synthase to state 3 respiration in Acanthamoeba castellanii mitochondria.. Acta Biochim Pol.

[50] Swida A, Czarna M, Woyda-Płoszczyca A, Kicinska A, Sluse FE, Jar-muszkiewicz W (2007). Fatty acid efficiency profile in uncoupling of Acan-thamoeba castellanii mitochondria.. J Bioenerg Biomembr.

[51] Woyda-Ploszczyca A, Jarmuszkiewicz W (2013). Hydroxynonenal-stimulated activity of the uncoupling protein in Acanthamoeba castellanii mitochondria under phosphorylating conditions.. Biol Chem.

[52] Zarkovic K (2003). 4-Hydroxynonenal and neurodegenerative diseases.. Mol Aspects Med.

[53] Antos-Krzeminska N, Kicinska A, Nowak W, Jarmuszkiewicz W (2023). Acanthamoeba castellanii Uncoupling Protein: A Complete Sequence, Activi-ty, and Role in Response to Oxidative Stress.. Int J Mol Sci.

[54] de Obeso Fernandez del Valle A, Scheckhuber CQ (2022). Superoxide Dismutases in Eukaryotic Microorganisms: Four Case Studies.. Antioxidants.

[55] Fang J, Beattie DS (2003). Alternative oxidase present in procyclic Trypanosoma brucei may act to lower the mitochondrial production of super-oxide.. Arch Biochem Biophys.

[56] Fang J, Beattie DS (2003). External alternative NADH dehydrogenase of Saccharomyces cerevisiae: a potential source of superoxide.. Free Radic Biol Med.

[57] Antos-Krzeminska N, Jarmuszkiewicz W (2014). External NAD(P)H Dehydrogenases in Acanthamoeba castellanii Mitochondria.. Protist.

[58] Clifton R, Millar AH, Whelan J (2006). Alternative oxidases in Arabidop-sis: a comparative analysis of differential expression in the gene family provides new insights into function of non-phosphorylating bypasses.. Biochim Biophys Acta.

[59] Ho LHM, Giraud E, Lister R, Thirkettle-Watts D, Low J, Clifton R, Howell KA, Carrie C, Donald T, Whelan J (2007). Characterization of the regulatory and expression context of an alternative oxidase gene provides insights into cyanide-insensitive respiration during growth and development.. Plant Physiol.

[60] Rasmusson AG, Fernie AR, Van Dongen JT (2009). Alternative oxidase: a defence against metabolic fluctuations?. Physiol Plant.

[61] de Obeso Fernández del Valle A, Scheckhuber CQ, Chavaro-Pérez DA, Ortega-Barragán E, Maciver SK (2023). mRNA Sequencing Reveals Up-regulation of Glutathione S-Transferase Genes during Acanthamoeba Encysta-tion.. Microorganisms.

[62] Cometa I, Schatz S, Trzyna W, Rogerson A (2011). Tolerance of naked amoebae to low oxygen levels with an emphasis on the genus Acanthamoeba.. Acta Protozool.

[63] Maciver SK, McLaughlin PJ, Apps DK, Piñero JE, Lorenzo-Morales J (2021). Opinion: Iron, Climate Change and the ‘Brain Eating Amoeba’ Naegleria fowleri.. Protist.

[64] Sifaoui I, -Yanes EC, Reyes-Batlle M, Rodríguez-Expósito RL, Bazzocchi IL, Jiménez IA, Piñero JE, Lorenzo-Morales J, Weaver LK (2021). High oxygen concentrations inhibit Acanthamoeba spp.. Parasitol Res.

[65] Khan NA (2006). Acanthamoeba: biology and increasing importance in human health.. FEMS Microbiol Rev.

[66] Rivera MA, Padhya TA (2002). Acanthamoeba: a rare primary cause of rhinosinusitis.. Laryngoscope.

[67] Cammaroto G, Astorga FJ, Navarro A, Olive T, Pumarola F (2015). Acanthamoeba rhinosinusitis: a paediatric case report and a review of the literature.. Int J Pediatr Otorhinolaryngol Extra.

[68] Fidalgo LM, Gille L (2011). Mitochondria and Trypanosomatids: Tar-gets and Drugs.. Pharm Res.

[69] Lobet E, Letesson J-J, Arnould T (2015). Mitochondria: A target for bacteria.. Biochem Pharmacol.

[70] Kilvington S, Price J (1990). Survival of Legionella pneumophila within cysts of Acanthamoeba polyphaga following chlorine exposure.. J Appl Bacte-riol.

[71] Cirillo JD, Falkow S, Tompkins LS (1994). Growth of Legionella pneu-mophila in Acanthamoeba castellanii enhances invasion.. Infect Immun.

[72] La Scola B, Raoult D (2001). Survival of Coxiella burnetii within fee-living amoeba Acanthamoeba castellanii.. Clin Microbiol Infect.

[73] Abd H, Saeed A, Weintraub A, Nair GB, Sandström G (2007). Vibrio cholerae O1 strains are facultative intracellular bacteria, able to survive and multiply symbiotically inside the aquatic free-living amoeba Acanthamoeba castellanii.. FEMS Microbiol Ecol.

[74] Lorenzo-Morales J, Coronado-Álvarez N, Martínez-Carretero E, Maciver SK, Valladares B (2007). Detection of four adenovirus serotypes within water-isolated strains of Acanthamoeba in the Canary Islands, Spain.. Am J Trop Med Hyg.

[75] Neilson JB, Ivey MH, Bulmer GS (1978). Cryptococcus neoformans: pseudohyphal forms surviving culture with Acanthamoeba polyphaga.. Infect Immun.

[76] Cirillo JD, Falkow S, Tompkins L, Bermudez L (1997). Interaction of Mycobacterium avium with environmental amoebae enhances virulence.. Infect Immun.

[77] Cirillo JD, Cirillo SL, Yan L, Bermudez LE, Falkow S, Tompkins LS (1999). Intracellular growth in Acanthamoeba castellanii affects monocyte entry mechanisms and enhances virulence of Legionella pneumophila.. Infect Immun.

[78] Larkin DF, Berry M, Easty DL (1991). In vitro corneal pathogenicity of Acanthamoeba.. Eye.

